# Functional Conducting Polymers via Thiol-ene Chemistry

**DOI:** 10.3390/bios2030305

**Published:** 2012-08-31

**Authors:** Kathleen E. Feldman, David C. Martin

**Affiliations:** Department of Materials Science and Engineering, University of Delaware, Newark, DE 19716, USA; E-Mail: katiefeldman0@gmail.com

**Keywords:** conducting polymer, thiol-ene, ProDOT, functionalization

## Abstract

We demonstrate here that thiol-ene chemistry can be used to provide side-chain functionalized monomers based on 3,4-propylenedioxythiophene (ProDOT) containing ionic, neutral, hydrophobic, and hydrophilic side chains. All reactions gave high yields and purification could generally be accomplished through precipitation. These monomers were polymerized either chemically or electro-chemically to give soluble materials or conductive films, respectively. This strategy provides for facile tuning of the solubility, film surface chemistry, and film morphology of this class of conducting polymers.

## 1. Introduction

Conducting polymers have found broad interest in recent years in applications as varied as photovoltaics [1], organic light emitting diodes [2], antistatic coatings [3], supercapacitors [4], biosensors [5], and neural electrodes [6,7,8]. The most commonly studied polymers include polypyrrole (PPy), poly(3-hexylthiophene) (P3HT), polyaniline (PANI), and poly(3,4-ethylenedioxythiophene) (PEDOT). The ultimate utility of these materials relies on the ability to optimize properties for a given application. To improve conductivity, for example, films are often annealed [9,10,11,12,13] (either thermally or in solvent vapor) or small molecule additives are incorporated [14,15,16]. Ultimately, however, the processability, surface chemistry, and functionality of conducting polymer films can only be tuned within a limited range without additional synthesis.

The appeal of conducting polymers as compared to inorganic semiconductors is often stated to arise from their solvent processability and chemical tunability. Additionally, the electrical and optical properties depend critically on interchain packing within a thin film. Tuning solubility and packing without disrupting conductivity along the backbone of the polymer requires functionalization of the side chains. Although numerous examples of functional conducting polymers have been shown, their synthesis is often tedious and the yields are low.

Because of the difficulty in developing soluble conducting polymers and the common requirement of a film geometry, electrochemical deposition onto conductive substrates is a widely utilized polymerization technique. In this case, functional groups can be incorporated into the film through small molecule or polymeric dopants [17,18,19]. As such films are electrochemically oxidized and reduced, it is possible for the dopants to be released since they are physically entrapped within rather than covalently bonded to the film. Although desirable in applications such as local drug delivery [20,21], there remains a need for reliable, stable film functionalization strategies. By incorporating the functional group into the monomer side chain, it is ensured that the functionality will remain in the film and not be lost upon electrochemical cycling.

In recent years, the rediscovery of the thiol-ene reaction has greatly expanded the scope of polymer functionalization chemistry [22,23]. This and related “click” reactions are generally highly tolerant to a wide range of functional groups and reaction conditions, proceed to high and often quantitative yields, and produce limited or no byproducts, leading to straightforward purification. Copper-catalyzed alkyne-azide coupling has been applied to both deposited films [24,25] and soluble [26,27] conducting polymers. As an alternative, thiol-ene is a radical-based reaction and as such is typically initiated via thermal or photochemical means and requires no metal catalyst. It has been shown highly useful in the synthesis of dendrimers [28], biodegradable hydrogels [29,30], and functionalized block and random copolymers [31]. Thiol-ene chemistry has also been applied to conducting polymers in the past; in their oxidized forms, PPy, PEDOT, and P3HT can react with various thiols to generate functionalized polymers [32,33]. The problem with this approach, however, is that it relies on backbone functionalization which can significantly disrupt or even destroy the electrical properties of the polymers. It is demonstrated here that thiol-ene chemistry can be used to synthesize functionalized monomers which can then be polymerized chemically or electrochemically to generate conducting polymers with tunable properties. 

## 2. Experimental Section

### 2.1. General Procedures and Materials

ProDOT-ene was synthesized according to the literature [34]. Sodium 3-mercapto-1-propanesulfonate was purchased from TCI America. Thioglycolic acid was purchased from Alfa Aesar. Thioglycerol, 2-ethylhexanethiol, and 3,4-propylenedioxythiophene were purchased from Sigma Aldrich. Propylene carbonate was dried and stored over type 4A molecular sieves. All other reagents and solvents were purchased from Fisher Scientific and used as received. Samples were irradiated using a UVP Black Ray UV Bench Lamp XX-15L, emitting 365 nm light at 15 W. NMR spectra (^1^H and ^13^C) were acquired on a Bruker DRX-400 spectrometer at room temperature. Chemical shifts are reported in parts per million, referenced to the solvent as internal standard (CHCl_3_: 7.24 ppm for ^1^H and 77.2 for ^13^C; DMSO: 2.50 for ^1^H and 77.2 ppm for ^13^C). FTIR spectra were collected on a Perkin Elmer Spectrum 100 spectrometer fitted with the Universal ATR accessory. Mass spectral data was collected on a Thermo LCQ spectrometer in the Mass Spectrometry Facility in the Chemistry and Biochemistry Department, University of Delaware. UV-Vis spectra were acquired on a Shimadzu UV-3600 spectrometer. Scanning electron microscopy images were acquired using a JSM-7400F field emission scanning electron microscope operating at 3 kV. Static water contact angles were measured by applying a 10 μL drop of deionized water to a film set on a leveled base; photographs were taken of the drops and their contact angles measured using ImageJ. Quoted values are the average of measurements from three separate areas of the film. Gel permeation chromatography (GPC) was conducted using a system comprising either two Waters Styragel (HR1 and HR4) columns and a Waters 2695 autosampler pump (Waters Corporation, Milford, MA, USA) with THF as the mobile phase (for P(EH-ProDOT)), or two Waters Ultrahydrogel (Linear and 250) columns and a Waters 515 HPLC pump with 80:20 0.10 M NaNO_3_/Acetonitrile as the mobile phase (for PAA-containing samples). All samples were run at a flow rate of 1.0 mL/min and detected with a Waters 2996 photodiode array and Waters 2414 refractive index detector in series. Waters Empower software was used to build calibration curves of narrow molecular weight polystyrene or poly(ethylene glycol) standards for data analysis. 

### 2.2. Synthesis of ProDOT-CO_2_H

ProDOT-ene (100 mg, 0.51 mmol) and thioglycolic acid (70 mg, 0.76 mmol) were added to a vial along with 0.1 wt% of 2,2-dimethoxy-2-phenylacetophenone (DMPA). The mixture was vortexed to ensure thorough mixing and dissolution of the initiator. The vial was placed under a UV lamp and irradiated for one hour. A small amount of methanol was added, and the product precipitated using water. The supernatant was decanted and the product redissolved in ethyl acetate, dried with MgSO_4_, and concentrated by evaporation to give a viscous oil (114 mg, 78%). ^1^H NMR (400 MHz, CDCl_3_): δ = 6.49 (s, 2H), 4.12 (dd, 2H), 3.92 (dd, 2H), 3.27 (s, 2H), 2.71 (t, 2H), 2.16 (m, 1H), 1.73 (m, 2H), 1.57 ppm (m, 2H). ^13^C NMR: δ = 175.8, 149.9, 105.8, 74.2, 42.1, 33.4, 32.7, 26.8, 26.3 ppm. FTIR: 3,109, 2,921, 2,864, 1,704, 1,560, 1,482, 1,451, 1,375, 1,295, 1,188, 1,130, 1,005, 850, 775 cm^–1^. Calculated: [M]^+^ C_12_H_16_O_4_S_2_ *m/z* = 288.05; found LC-MS: [2M]^+^ *m/z* = 573.5.

### 2.3. Synthesis of ProDOT-NH_2_

Cysteamine HCl (86 mg, 0.76 mmol) and a minimum of methanol were added to a vial and heated to dissolve the thiol. ProDOT-ene (100 mg, 0.51 mmol) and 0.1 wt% of 2,2-dimethoxy-2-phenylacetophenone (DMPA) were added, and the mixture sparged with argon for five minutes. The vial was placed under a UV lamp and irradiated for one hour. Diethyl ether was added to precipitate ProDOT-NH_3_Cl, which was filtered and dried under vacuum. The dried product was dissolved in water (2 mL) and 10% NaOH was added to neutralize the ammonium side chains. The aqueous phase was extracted using ethyl acetate (3 × 5 mL). The combined organic phases were dried with MgSO_4_ and concentrated by evaporation to give a viscous oil (121 mg, 87%). ^1^H NMR (400 MHz, CDCl_3_): δ = 6.48 (s, 2H), 4.11 (dd, 2H), 3.90 (dd, 2H), 2.88 (br, 2H), 2.63 (t, 2H), 2.54 (t, 2H), 2.15 (m, 1H), 1.82 (br, 2H), 1.68 (m, 2H), 1.55 (m, 2H). ^13^C NMR: δ = 149.9, 105.7, 74.3, 42.1, 40.9, 36.1, 31.7, 27.1, 27.0 ppm. FTIR: 3,104, 2,921, 2,858, 1,561, 1,484, 1,454, 1,377, 1,192, 1,013, 854, 780 cm^–1^. Calculated: [M]^+^ C_12_H_19_NO_2_S_2_ *m/z* = 273.09; found LC-MS: [M^+^H^+^] *m/z* = 274.3.

### 2.4. Synthesis of ProDOT-SO_3_Na

Sodium 3-mercapto-1-propanesulfonate (120 mg, 0.68 mmol) and a minimum of methanol were added to a vial and heated to dissolve the thiol. ProDOT-ene (200 mg, 1.02 mmol) and 0.1 wt% of 2,2-dimethoxy-2-phenylacetophenone (DMPA) were added, and the mixture sparged with argon for five minutes. The vial was placed under a UV lamp and irradiated for one hour. Diethyl ether was added to precipitate the product, which was filtered and dried under vacuum to give a white powder (230 mg, 91%). ^1^H NMR (400MHz, CD_3_OD): δ = 6.52 (s, 2H), 4.08 (dd, 2H), 3.87 (dd, 2H), 2.92 (m, 2H), 2.66 (t, 2H), 2.57 (t, 2H), 2.13 (m, 1H), 2.05 (m, 2H), 1.70 (m, 2H), 1.55 (m, 2H). ^13^C NMR: δ = 151.9, 106.8, 75.9, 51.6, 43.8, 32.7, 31.8, 28.2, 28.1, 26.3 ppm. FTIR: 3,451, 3,107, 2,924, 2,862, 1,732, 1,640, 1,560, 1,484, 1,452, 1,376, 1,184, 1,049, 1,008, 853, 776, 737 cm^−1^. Calculated: [M]^+^ C_13_H_19_NaO_5_S_3_ *m/z* = 374.03; found LC-MS: [2M]^++^ *m/z* = 748.8.

### 2.5. Synthesis of ProDOT-Glycerol

ProDOT-ene (100 mg, 0.51 mmol) and thioglycerol (83 mg, 0.76 mmol) were added to a vial along with 0.1 wt% of 2,2-dimethoxy-2-phenylacetophenone (DMPA). A minimum of methanol was added to dissolve the reactants and the mixture sparged with argon for five minutes. The vial was placed under a UV lamp and irradiated for one hour. The product was then precipitated using water. The supernatant was decanted and the product redissolved in ethyl acetate, dried with MgSO_4_, and concentrated by evaporation to give a viscous oil (100 mg, 65%). ^1^H NMR (400 MHz, CDCl_3_): δ = 6.49 (s, 2H), 4.11 (dd, 2H), 3.92 (dd, 2H), 3.81 (m, 1H), 3.76 (dd, 1H), 3.57 (dd, 1H), 2.72 (dd, 1H), 2.63 (d, 1H), 2.58 (t, 2H), 2.54 (br, 2H), 2.15 (m, 1H), 1.70 (m, 2H), 1.55 (m, 2H). ^13^C NMR: δ = 149.9, 105.8, 74.2, 69.9, 65.3, 42.1, 35.7, 32.3, 27.0, 26.9 ppm. FTIR: 3,383, 3,107, 2,917, 2,865, 1,559, 1,483, 1,452, 1,375, 1,190, 1,007, 851, 776 cm^–1^. Calculated: [M]^+^ C_13_H_20_O_4_S_2_ *m/z* = 304.08; found LC-MS: [M^+^H^+^] *m/z* = 305.3.

### 2.6. Synthesis of Ethylhexyl-ProDOT (EH-ProDOT)

ProDOT-ene (250 mg, 1.28 mmol) and 2-ethylhexanethiol (330 μL, 1.90 mmol) were added to a vial along with 0.1 wt% of 2,2-dimethoxy-2-phenylacetophenone (DMPA). The mixture was vortexed to ensure thorough mixing and dissolution of the initiator. The vial was placed under a UV lamp and irradiated for one hour. The product was purified by column chromatography, eluting with 10% ethyl acetate/90% hexanes, to give a clear oil (325 mg, 74%). ^1^H NMR (400 MHz, CDCl_3_): δ = 6.49 (s, 2H), 4.13 (dd, 2H), 3.90 (dd, 2H), 2.51 (q, 4H), 2.17 (m, 1H), 1.68 (m, 2H), 1.60-1.20 (m, 11H), 0.91 (t, 3H), 0.88 (t, 3H). ^13^C NMR: δ = 150.0, 105.7, 74.4, 42.2, 39.3, 36.8, 32.8, 32.4, 28.9, 27.1, 25.5, 23.0, 14.1, 10.8 ppm. FTIR: 3,108, 2,957, 2,924, 2,858, 1,560, 1,484, 1,454, 1,375, 1,190, 1,043, 1,012, 851, 773 cm^–1^. Calculated: [M]^+^ C_18_H_30_O_2_S_2_ *m/z* = 342.17; found LC-MS: [M^+^H^+^] *m/z* = 343.5.

### 2.7. Poly(EH-ProDOT)

FeCl_3_ (142 mg, 0.87 mmol) was suspended in 2 mL of CHCl_3_ with stirring. A solution of EH-ProDOT (100 mg, 0.29 mmol) dissolved in 1 mL of CHCl_3_ was added dropwise, upon which the solution immediately turned dark red. After stirring for 18 h at room temperature, excess methanol was added to precipitate the polymer and wash away any remaining FeCl_3_. The resulting solid was filtered and washed with methanol, then dried under vacuum to give 85 mg (85% yield) of a purple powder.

### 2.8. Poly(ProDOT-SO_3_Na)

FeCl_3_ (165 mg, 1 mmol) was suspended in 5 mL of CHCl_3_. A solution of ProDOT-SO_3_Na (100 mg, 0.29 mmol) suspended in 10 mL of CHCl_3_ was added dropwise. After stirring for 18 h at room temperature, excess methanol was added to precipitate the polymer and wash away any remaining FeCl_3_. The resulting dark solid was dissolved in 1 M NaOH and stirred for two days to exchange iron counterions for sodium. Excess methanol was added to precipitate the polymer, and it was then redissolved in deionized water and dialyzed for two days against deionized water using a 1,000 g/mol cutoff membrane. The resulting solution was freeze-dried to give 68 mg (68% yield) of a brown powder.

### 2.9. Electropolymerization Procedure

Polymer films were electrochemically deposited under galvanostatic conditions (0.5 mA/cm^2^) with an Autolab PGstat12 Potentiostat/Galvanostat (EcoChemie) using a three electrode cell. Gold coated silicon wafers (Platypus Technologies) served as the working electrodes and a platinum wire as the counter electrode, with a Ag/Ag^+^ wire as the pseudoreference electrode. Deposition solutions consisted of monomer (50 mM) and tetrabutylammonium perchlorate (TBAP, 100 mM) dissolved in propylene carbonate. Deposited films were characterized by cyclic voltammetry in 1× phosphate buffered saline using a three electrode arrangement with Ag/AgCl reference electrode and platinum wire counter electrode. Measurements were taken at 120 mV/s between −0.6 V and +0.8 V for 100 cycles.

## 3. Results and Discussion

**Scheme 1:**
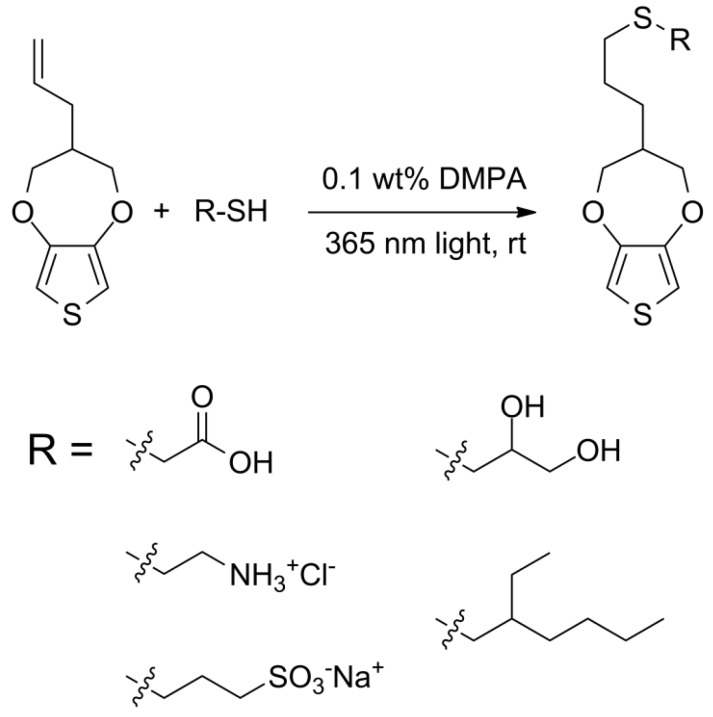
Synthesis of side-chain functionalized ProDOT derivatives.

Using the previously developed ProDOT-ene [34,35] as a precursor, a library of functional monomers was synthesized in high yield under benign reaction conditions (Scheme 1). All reactions were conducted using photochemical initiation (365 nm UV light) with 2,2-dimethoxyacetophenone (DMPA) as initiator. In the case of liquid thiols miscible with ProDOT-ene, such as thioglycolic acid, the reaction was complete within 30 minutes of irradiation with 365 nm UV light (as determined by the disappearance of alkene peaks between 5.0 and 6.0 ppm in ^1^H NMR). When solid or immiscible liquid thiols were used (e.g., cysteamine HCl or thioglycerol), a small amount of solvent was required, and the reaction mixture was sparged with argon before irradiation to ensure complete reaction. Purification was typically accomplished by precipitation using appropriate solvents, but in some cases extraction and washing or column chromatography was required. Isolated yields of 80–95% were typical, with the loss primarily attributable to incomplete recovery during purification.

### 3.1. Electrochemical Polymerization of Functional Monomers

**Figure 1:**
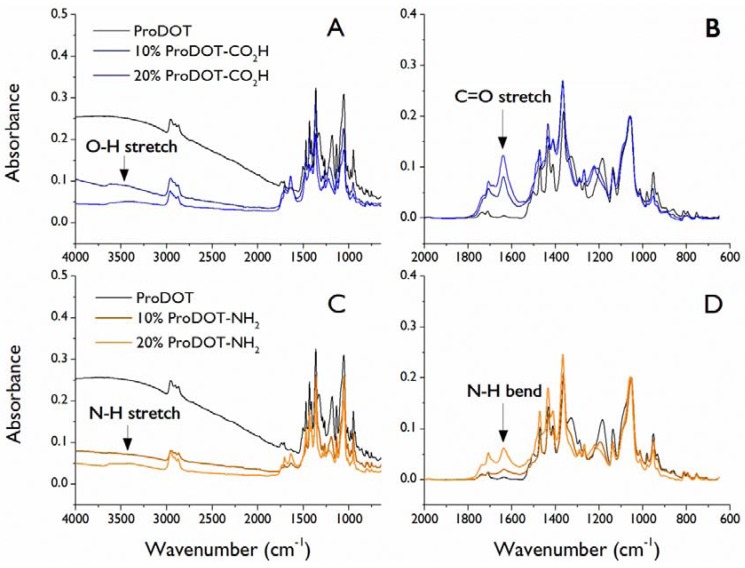
FTIR spectra of electrochemically deposited films. Deposition solutions contained a total monomer concentration of 50 mM and either 100% ProDOT, 90% ProDOT + 10% functionalized ProDOT, or 80% ProDOT + 20% functionalized ProDOT. (**A**) Full spectra and (**B**) fingerprint region of ProDOT-CO_2_H-containing films; (**C**) full spectra and (**D**) fingerprint region of ProDOT-NH_2_-containing films.

Several of the functional monomers were tested for their ability to be electrochemically polymerized. This strategy allows for conformal films to be generated on conductive substrates while tuning the surface chemistry through the side chain functionality. The monomers chosen for electrochemical polymerization included ProDOT-CO_2_H, ProDOT-NH_2_, and ProDOT-glycerol. Although deposition of films of pure functional monomer was found to be difficult, each could be co-deposited with non-functionalized 3,4-propylenedioxythiophene (ProDOT) at levels up to 20 mol% (functional monomer fraction in deposition solution). FTIR spectra of the deposited films clearly demonstrate the incorporation of functional monomer; Figure 1 shows such data for films deposited from solutions containing 0, 10, and 20 mol% ProDOT-CO_2_H. The spectra show peaks at 1,640 cm^–1^ (C=O stretch) and 3,415 cm^–1^ (OH stretch) characteristic of the carboxylic acid containing monomer. Baseline subtraction of the region between 2,000 and 650 cm^–1^ and normalization to the peak at 1,060 cm^–1^ (C–O stretch from dioxypropylene ring; intensity should be insensitive to oxidation state of polymer) demonstrates that more functional monomer is incorporated into the film with an increase in the relative concentration in the deposition solution, since the intensity of the carbonyl stretch increases. Similarly, films deposited from solutions containing ProDOT-NH_2_ show peaks at 1,632 cm^–1^ (N–H bend) and 3,450 cm^–1^ (N–H stretch) that increase in intensity with an increase in functional monomer content in the deposition solution. No distinguishing peaks were seen in films containing ProDOT-glycerol or ProDOT-SO_3_Na. Changes in hydrophilicy also indicate that functional monomer was incorporated into the films. With 20 mol% comonomer in the deposition solution, both ProDOT-CO_2_H and ProDOT-NH_2_ containing films showed a decrease in static water contact angle from 59° to 43° as compared to pure ProDOT films, while 20 mol% ProDOT-glycerol solutions produced films with contact angles of 50° (although changes in film morphology as shown below can also affect contact angles). Data could not be obtained for films containing ProDOT-SO_3_Na because they were immediately wetted and swollen by the water droplets.

### 3.2. Morphology of Deposited Films

**Figure 2:**
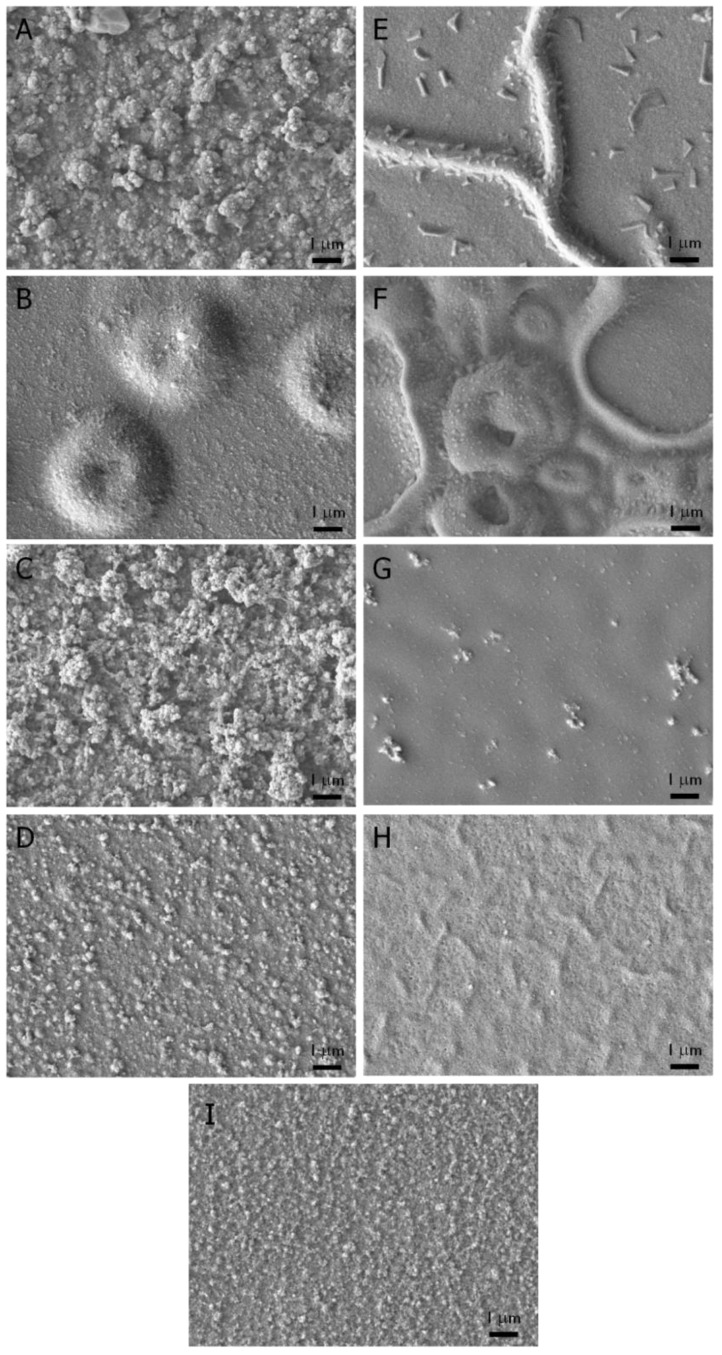
SEM micrographs of films electrochemically deposited from solutions of (**A**) 10% ProDOT-CO_2_H + 90% ProDOT; (**B**) 20% ProDOT-CO_2_H + 80% ProDOT; (**C**) 10% ProDOT-NH_2_ + 90% ProDOT; (**D**) 20% ProDOT-NH_2_ + 80% ProDOT; (**E**) 10% ProDOT-glycerol + 90% ProDOT; (**F**) 20% ProDOT-glycerol + 80% ProDOT; (**G**) 10% ProDOT-SO_3_Na + 90% ProDOT; (**H**) 20% ProDOT-SO_3_Na + 80% ProDOT and (**I**) ProDOT. Composition refers to the relative molar percentage in deposition solution. In all cases total monomer concentration was 50 mM in propylene carbonate with 100 mM TBAP as supporting electrolyte/counterion.

The surface morphology of electrochemically deposited films has a significant effect on their redox properties; greater surface area tends to reduce electrochemical impedance, a critical property for applications in neural interfaces [36]. This is beneficial in devices such as neural prosthetics that critically rely on efficient charge transport across the film interface. Additionally, it has been shown that roughness can have a dramatic effect on the ability of cells to adhere to functional surfaces [37,38]. Scanning electron microscopy characterization of the electrochemically deposited films containing ProDOT-CO_2_H showed an increase in roughness with 10% comonomer in the feed (Figure 2(A)). At 20% comonomer (Figure 2(B)), the local roughness (on the submicron scale) decreased, but large bubbles of several to tens of microns in diameter were seen to cover most of the film. Incorporation of the carboxylic acid containing monomer into the film presumably allows it to swell in the deposition solution to a greater extent than pure ProDOT films, leading to bubbling as the film thickness increases. Similarly, large wrinkles were seen in films deposited from 10% ProDOT-glycerol solution (Figure 2(E)), and bubbles appeared in films deposited from 20% ProDOT-glycerol solution (Figure 2(F)). Films generated from ProDOT-NH_2_-containing solutions (Figure 2(C,D)) had a similar morphology to pure ProDOT films (Figure 2(I)), while ProDOT-SO_3_Na-containing films were relatively smooth (Figure 2(G,H)).

### 3.3. Electrochemical Behavior of Functional Films

**Figure 3:**
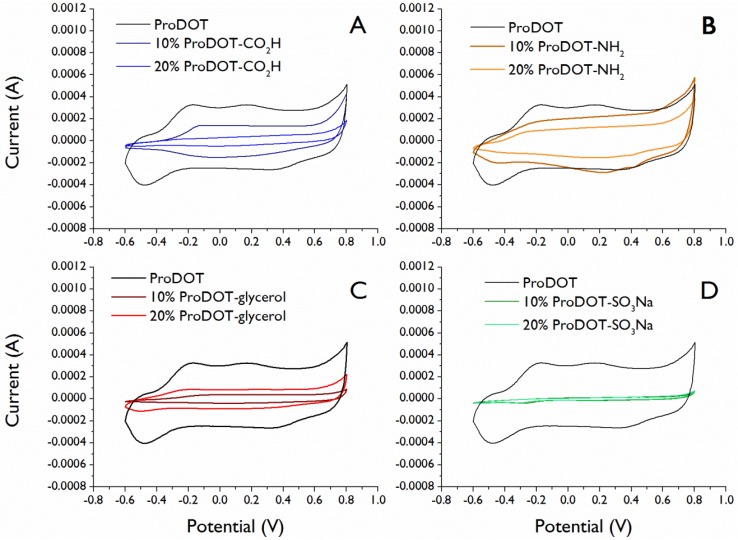
Cyclic voltammetry data for functional ProDOT films electrochemically deposited from solutions of (**A**) 10% ProDOT-CO_2_H + 90% ProDOT and 20% ProDOT-CO_2_H + 80% ProDOT, (**B**) 10% ProDOT-NH_2_ + 90% ProDOT and 20% ProDOT-NH_2_ + 80% ProDOT, (**C**) 10% ProDOT-glycerol + 90% ProDOT and 20% ProDOT-glycerol + 80% ProDOT, and (**D**) 10% ProDOT-SO_3_Na + 90% ProDOT and 20% ProDOT-SO_3_Na + 80% ProDOT. Data are shown from cycle #50 after the behavior of each film stabilized.

The electrochemically deposited films were characterized by cyclic voltammetry to demonstrate their conductivity and compare to pure ProDOT films. As seen in Figure 3, the oxidation and reduction potentials of the films containing functional monomer are not substantially changed as compared to ProDOT films, yet in all cases the electrochemical activity decreased with increasing comonomer in the feed solution. In the case of ProDOT-SO_3_Na, much of the film appeared to wash away upon electrochemical evaluation in aqueous solutions due to the high water solubility of the functional monomer. The decrease in electrical activity is likely due in part to the changes in film morphology seen by SEM (Figure 2). Although conductivity is maintained by functionalizing the side chain rather than the polymer backbone, the change in monomer solubility still affects the electrochemical properties of films in an indirect way.

### 3.4. Synthesis of Soluble Conducting Polymers

**Figure 4:**
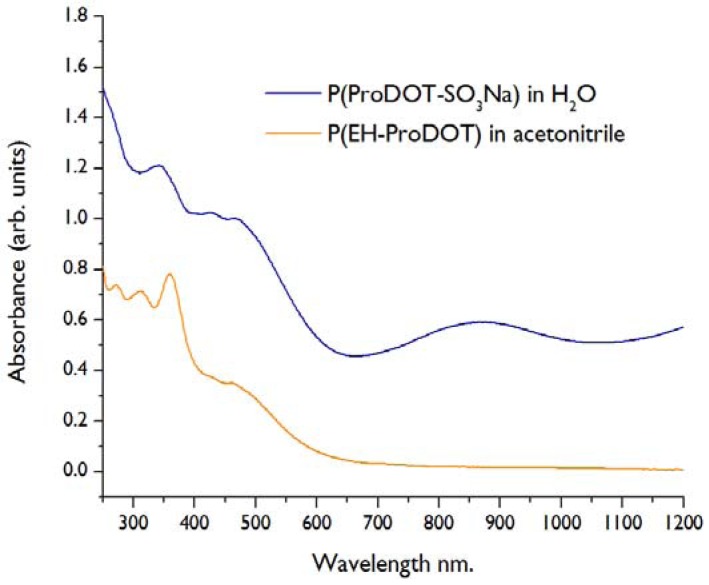
UV-Vis spectra of water and organic soluble polymers.

Monomer functionalization via thiol-ene chemistry is also an effective strategy to synthesize soluble polymers. The utility of soluble conducting polymers is demonstrated by the significant commercial success of PEDOT/PSS dispersions (Clevios™ P), which can be spin coated to give transparent conductive layers on a variety of substrates. Using the current strategy, ProDOT-SO_3_Na was polymerized in solution with FeCl_3_, precipitated with methanol, and purified by dialysis to give a fully water soluble material. Similarly, ethylhexyl-ProDOT was polymerized in CHCl_3_ solution using FeCl_3_ and precipitated with methanol to give a polymer soluble in CHCl_3_, THF, and acetonitrile. UV-Vis spectra (Figure 4) of the two polymers show broad absorption characteristic of the partially oxidized state of the polymers.

GPC chromatograms were collected for each polymer under appropriate conditions. P(ProDOT-SO_3_Na) had a number average molecular weight M_n_ of 2500 (PEG equivalent) and PDI of 2.0. P(EH-ProDOT) was found to consist primarily of low molecular weight oligomeric material (M_n_ less than 1,500 g/mol in PS equivalents). It is likely that the ethylhexyl side chain is not bulky enough to allow for significant polymer solubility; doubly substituted derivatives [35] might provide greater solubility and allow for higher molecular weight materials to be synthesized. 

## 4. Conclusions

In summary, it has been shown that thiol-ene chemistry is a highly effective and simple method enabling the rapid synthesis of a broad array of functional conducting polymers. Monomers containing biologically relevant side chains or groups available for further functionalization could be electrochemically polymerized to give conductive films displaying such functionality on the surface. The electrical activity of the films decreased with increasing incorporation of functional monomer, likely due to changes in film morphology, indicating that for future applications a balance must be struck between providing surface functionality and maintaining desirable electrochemical behavior. Polymers soluble in either water or organic solvents could also be synthesized by starting with monomers containing strongly acidic or branched alkyl side chains, respectively. Each of these developments is expected to broaden the range of both existing and new applications for conducting polymers.
